# Incidence of early complications requiring treatment plan changes after vitreoretinal surgery: a single-center study in South Korea

**DOI:** 10.1186/s12886-023-03030-z

**Published:** 2023-06-19

**Authors:** Ji Hyun Yoon, Jae Hui Kim, Chul Gu Kim, Jong Woo Kim

**Affiliations:** Department of Ophthalmology, Kim’s Eye Hospital, #156 Youngdeungpo-dong 4ga, Youngdeungpo-gu, Seoul, 150-034 South Korea

**Keywords:** Vitrectomy, Treatment plan, Standardized care, Tamponade, Surgeon’s experience, Postoperative complications, Patient care

## Abstract

**Background:**

Information regarding incidence of treatment plan changes may be useful when discussing postoperative treatment plans for patients. Moreover, it may help establish a standardized postoperative treatment plan. This study aimed to evaluate the incidence of early complications requiring treatment plan changes in patients following vitreoretinal surgery and investigate its risk factors.

**Methods:**

This single-center retrospective study included 465 patients who had undergone vitreoretinal surgery. The reasons, incidence, and timing of treatment plan changes within 14 days of surgery were identified. Potential factors associated with the changes, such as patient demographics, surgeon’s experience, diagnoses, and type of surgery were also analyzed.

**Results:**

The treatment plan was changed in 76 patients (16.3%) at a mean of 4.0 ± 3.2 days after vitreoretinal surgery. The reasons for the plan changes were increased intraocular pressure (IIOP) in 66(86.8%), intraocular inflammation in 2(2.6%), corneal edema in 3(3.9%), leakage from the sclerotomy wound in 3(3.9%) patients, and combined IIOP and intraocular inflammation in 2(2.6%). The date of discharge was postponed because of treatment plan changes in 17 patients (22.4%). The incidence of plan changes was higher in patients who underwent gas or oil tamponade (*P* < 0.001) and those who underwent surgery performed by less experienced surgeons (*P* = 0.034).

**Conclusions:**

Treatment plan was changed in 16.3% of patients after vitreoretinal surgery. The risk of treatment plan changes was associated with the surgeon’s experience in vitreoretinal surgery and the type of surgery. These results should be considered when establishing standardized care plans for patients who require vitreoretinal surgery.

## Intruduction

Modern vitreoretinal surgery is safe and effective in treating various retinal disorders, including rhegmatogenous retinal detachment (RRD), [1] complications of diabetic retinopathy, [2] macular hole (MH), [3] and epiretinal membrane (ERM) [4]. However, since vitreoretinal surgery is invasive, postoperative complications, such as increased (2.5–61.5%) or decreased (1.8–13.1%) intraocular pressure (IOP), [5–10] corneal edema (0.80–1.1%) [7, 11], intraocular inflammation (0.25–26%) [5, 12], or retinal detachments (1.2–1.7%) may occur in patients [11]. Therefore, the postoperative care plan for patients may be changed according to the type and severity of these complications [12].

The incidence of postoperative complications varies substantially among patients who undergo different surgical procedures [13]. Accordingly, the incidence of changes in treatment plans may also differ. Information regarding these changes may be useful when discussing postoperative treatment plans for patients. In addition, it may help establish a standardized postoperative care system [14]. To date, there is limited knowledge regarding this topic.

Herein, we investigated the incidence of early complications requiring changes in the treatment plan in patients who had undergone vitreoretinal surgery. Additionally, the factors associated with the risk of plan changes were evaluated.

## Methods

This retrospective study was conducted at a single center (Kim’s Eye Hospital, Seoul, South Korea, #2022-05-009). The study was approved by the Institutional Review Board of Kim’s Eye Hospital and conducted in accordance with the tenets of the Declaration of Helsinki. The need for informed consent was waived by the Institutional Review Board (Kim’s Eye Hospital IRB).

## Patients

We secured a list of patients who underwent vitrectomy with or without gas or silicone oil tamponade, scleral buckling, scleral encircling, or scleral fixation of intraocular lens (IOL) at our institution between January 2021 and December 2021. Patients with the following diagnoses were included: RRD, ERM, MH, DR, vitreous hemorrhage (VH) other than DR, IOL dislocation, or lens dislocation. All vitrectomies were performed with transconjunctival sutureless vitrectomy technique using 23-gauge or 25-gauge device.

## Outcome measures

Patients who experienced changes in the treatment plan within 14 days after surgery due to postoperative complications were identified. The timing, reasons, and details of these changes were also identified. The incidence of the changes in treatment plan was compared among the following categories: (1) age; (2) sex; (3) diabetes mellitus; (4) hypertension; (5) surgeon’s experience in vitreoretinal surgery (≥ 6 vs. < 6 years); (6) diagnosis: RRD vs. ERM vs. MH vs. DR vs. VH other than DR vs. IOL dislocation or lens dislocation; (7) type of surgery: vitrectomy without tamponade vs. vitrectomy with tamponade vs. scleral buckling or encircling only vs. scleral fixation; and (8) combined cataract surgery. When there was more than one episode of plan change, only the first episode of plan change was included in this study.

### Statistical analysis

Data are presented as the mean ± standard deviation or number (%), where applicable. Statistical analyses were performed using the IBM SPSS Statistics for Windows (version 21.0; IBM Corp., Armonk, NY, USA). The visual acuities were represented as logarithm of the minimum angle of resolution (logMAR) values. The visual acuities for counting finger and hand motion were converted to logMAR values of 2 and 3, respectively. Between patients with and without treatment plan changes, the age was compared using the independent samples t-test. Diabetes mellitus, hypertension, history of vitreoretinal surgery, surgeon’s experience in vitreoretinal surgery, diagnosis, type of surgery, and combined cataract surgery were compared between the two groups using the chi-square test. The incidence of complications among patients requiring treatment plan changes according to the type of surgery and the timing at which the complications occurred was analyzed using the chi-square or Fisher’s exact test. Statistical significance was set at *P* < 0.05.

## Results

### Demographics

A total of 465 patients were included in the study (Table 1). Two hundred and seventy-three patients were men (58.7%) and 192 were women (41.3%). Their mean age was 58.20 ± 13.52 years; 15 (3.2%) of them had a history of vitreoretinal surgery, 133 (28.6%) had diabetes mellitus, and 205 (44.1%) had hypertension. The logMAR preoperative best-corrected visual acuity was 0.98 ± 1.01 and the mean axial length was 24.42 ± 1.80 mm.

**Table 1:** Baseline characteristics and type of surgery of patients included in the study (*n* = 465)

Characteristics	Values
Age, years	58.20 ± 13.52
Sex	
Men	273 (58.7%)
Women	192 (41.3%)
Diabetes mellitus	133 (28.6)
Hypertension	205 (44.1)
Best-corrected visual acuity, logMAR	0.98 ± 1.01
Axial length, mm	24.42 ± 1.80
History of vitreoretinal surgery	15 (3.2)
Surgeon’s experience in vitreoretinal surgery	
≥ 6 years	334 (71.8)
< 6 years	131 (28.2)
Diagnosis	
Epiretinal membrane	86 (18.5)
DR	76 (16.3)
With VH	65 (14.0)
Without VH	11 (2.4)
Macular hole	53 (11.4)
Rhegmatogenous retinal detachment	158 (34.0)
VH other than DR	26 (5.6)
RVO	17 (3.7)
AMD	5 (1.1)
Others^a^	4 (0.9)
IOL dislocation or lens dislocation	66 (14.2)
Type of surgery	
Vitrectomy without tamponade	174 (37.4)
Vitrectomy with tamponade	184 (39.6)
Gas	156 (33.5)
Silicone oil	28 (6.0)
Vitrectomy with IOL scleral fixation	62 (13.3)
Scleral buckling or encircling only	45 (9.7)
Combined cataract surgery	95 (20.4)

Abbreviation: *IOL *Intraocular lens, *logMAR *Logarithm of the minimum angle of resolution

^a^Central retinal artery occlusion, trauma, and age-related macular degeneration

The diagnosis was ERM in 86 patients (18.5%), diabetic retinopathy in 76 patients (16.3%), MH in 53 patients (11.4%), RRD in 158 patients (34.0%), VH other than DR in 26 patients (5.6%), and IOL dislocation or lens dislocation in 66 patients (14.2%). One hundred seventy-four patients underwent vitrectomy without tamponade, 184 underwent vitrectomy with tamponade using either gas (*n* = 156) or silicone oil (*n* = 28), 62 underwent vitrectomy with IOL scleral fixation, and 45 underwent scleral buckling or encircling only. Fifteen surgeons performed the surgery. Three hundred thirty-four cases (71.8%) were operated on by surgeons with ≥ 6 years of experience in vitreoretinal surgery. Surgeons with < 6 years of experience performed the procedure in the remaining 131 cases (28.2%).

### Treatment plan changes

**Fig. 1:**
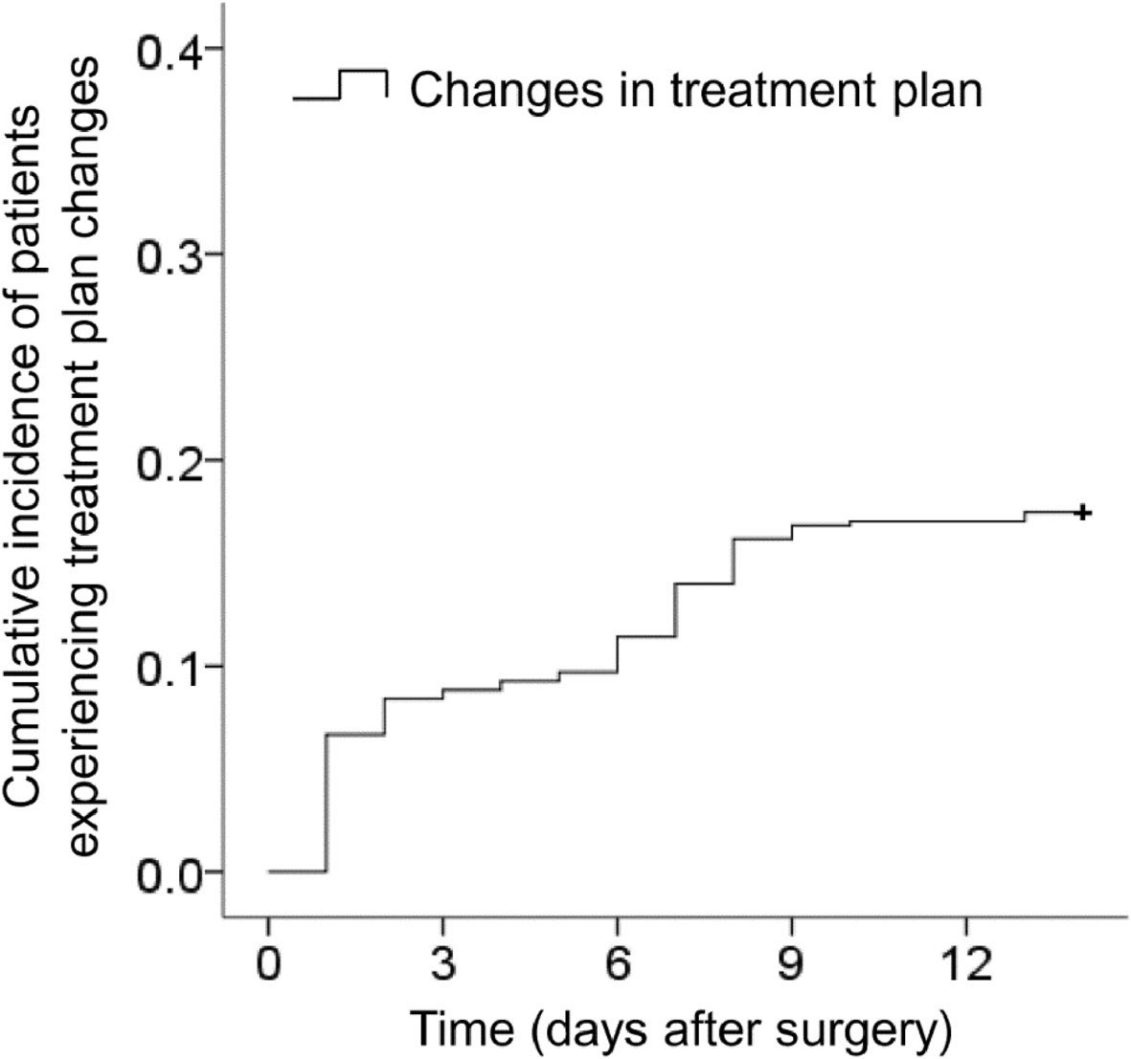
Kaplan–Meier graph showing the cumulative incidence of patients experiencing treatment plan changes after surgery

Among the 465 patients, the treatment plan was changed within 14 days after surgery in 76 patients (16.3%). The mean period between the surgery and plan changes was 4.0 ± 3.2 days (Fig. 1). The plan was changed 1 day after the surgery in 33 patients (43.4%), between 2 and 7 days in 29 patients (38.1%), and between 8 and 14 days in 14 patients (18.4%). The reasons for the plan changes were increased intraocular pressure in 66 (86.8%), intraocular inflammation in 2 (2.6%), corneal edema in 3 (3.9%), leakage from the sclerotomy wound in 3 (3.9%) patients, and combined increased intraocular pressure and intraocular inflammation in 2 (2.6%). Table 2 summarizes the reasons for the plan changes according to the type of surgery.

**Table 2:** Incidence of complications requiring treatment plan changes according to the type of surgery

Type of surgery	Increase in IOP	Intraocular inflammation	Hypotony	Corneal edema
Vitrectomy without tamponade (*n* = 174)	7 (4.0)	0 (0.0)	0 (0.0)	1 (0.6)
Vitrectomy with tamponade (*n* = 184)	52 (28.3)	4 (2.2)	2 (1.1)	0 (0.0)
Vitrectomy with IOL scleral fixation (*n* = 62)	7 (11.3)	0 (0.0)	1 (1.6)	2 (3.2)
Scleral buckling or encircling only (*n* = 45)	2 (4.4)	0 (0.0)	0 (0.0)	0 (0.0)
*P*-value	< 0.001*	0.214^†^	0.106^†^	0.054^†^

Abbreviations: *IOL *Intraocular lens, *IOP *Intraocular pressure

*Statistical analysis was performed using the chi-square test

† Statistical analyses were performed using the Fisher’s exact test

The specific contents of the treatment plan changes were as follows: changes in the frequency of administration of topical drugs only = 4, adding another topical drug = 66, changes in the usage of systemic drugs with or without changes in the usage of topical drugs = 10, and performing invasive procedures, such anterior chamber tapping or intravitreal injection = 5 with or without changes in drug therapy. All four intraocular inflammation cases were well-controlled after changes in the frequency of administration of topical drugs or the addition of other topical drugs. None of the patients had infectious endophthalmitis. Table 3 summarizes the timing of the treatment plan changes according to the reasons. Among the 76 patients, the date of discharge was postponed owing to treatment plan changes in 17 patients (21.8%). The reason was an increase in IOP in 13 patients, intraocular inflammation in 1, hypotony in 1, and combined increased intraocular pressure and intraocular inflammation in 2.

**Table 3:** Timing at which the complications requiring treatment plan changes was identified

Reasons of treatment plan changes	Postoperative days			
	≤ 1	> 1, ≤ 7	> 7, ≤ 14	*P*-value
Increase in IOP (*n* = 66)	25 (37.9)	28 (42.4)	13 (19.7)	0.827*
Intraocular inflammation (*n* = 2)	2 (100.0)	0 (0)	0 (0)	0.663^†^
Hypotony (*n* = 3)	2 (66.7)	1 (33.3)	0 (0)	0.213^†^
Corneal edema (*n* = 3)	2 (66.7)	0 (0)	1 (33.3)	0.399^†^
Increase in IOP + Intraocular inflammation (*n* = 2)	2 (100.0)	0 (0)	0 (0)	0.663^†^

Abbreviations: *IOP *Intraocular pressure

*Statistical analysis was performed using the chi-square test

† Statistical analyses were performed using the Fisher’s exact test

### Factors associated with treatment plan changes

Table 4 summarizes the results of comparisons between patients with and without changes in the treatment plan. A history of vitreoretinal surgery (*P* = 0.001), surgeon’s experience in vitreoretinal surgery (*P* = 0.034), diagnosis (*P* < 0.001), and type of surgery (*P* < 0.001) were associated with different incidences of plan changes. More specifically, the incidence was relatively higher in patients with a history of vitreoretinal surgery (46.7%), those operated on by surgeons with < 6 years of experience in vitreoretinal surgery (22.1%), those diagnosed with RRD (23.4%) or MH (26.4%), and those who underwent vitrectomy with tamponade (30.4%).

**Table 4:** Comparisons of incidence of treatment plan changes between patients with different characteristics (*n* = 465)

Characteristics	Plan changes (+) (*n* = 76)	Plan changes (–) (*n* = 389)	*P*-value
Age, years	57.91 ± 13.0	58.26 ± 13.6	0.832*
Diabetes mellitus			0.111†
Yes	16 (12.0)	117 (88.0)	
No	60 (18.1)	272 (81.9)	
Hypertension			0.256†
Yes	38 (18.5)	167 (81.5)	
No	38 (14.6)	222 (85.4)	
History of vitreoretinal surgery			0.001†
Yes	7 (46.7)	8 (53.3)	
No	69 (15.3)	381 (84.7)	
Surgeon’s experience in vitreoretinal surgery			0.034†
≥ 6 years	47 (14.1)	287 (85.9)	
< 6 years	29 (22.1)	102 (77.9)	
Diagnosis			< 0.001†
Rhegmatogenous retinal detachment	37 (23.4)	121 (76.6)	
Epiretinal membrane	3 (3.5)	83 (96.5)	
Macular hole	14 (26.4)	39 (73.6)	
DR	9 (11.8)	67 (88.2)	
VH other than DR	2 (7.7)	24 (92.3)	
IOL dislocation or lens dislocation	11 (16.7)	55 (83.3)	
Type of surgery			< 0.001^†^
Vitrectomy without tamponade	7 (4.0)	166 (96.0)	
Vitrectomy with tamponade	56 (30.4)	128 (69.6)	
Vitrectomy with IOL scleral fixation	11 (17.5)	52 (82.5)	
Scleral buckling or encircling only	2 (4.4)	43 (95.6)	
Combined cataract surgery			0.432†
Combined operation	13 (13.7)	82 (86.3)	
Non-combined operation	63 (17.0)	307 (83.0)	

Data are presented as mean ± standard deviation or no. (%) where applicable

Abbreviation: *IOL *Intraocular lens

*Statistical analysis was performed using the independent sample t-test

†Statistical analysis was performed using the chi-squared test

## Discussion

In the present study, changes in the treatment plan after vitreoretinal surgery were noted in 16.3% of patients at a mean of 4.0 days after surgery. The most prevalent reason was an increase in IOP, followed by intraocular inflammation, hypotony due to leakage from the sclerotomy wound, and corneal edema. In most patients, the events causing plan changes were well-controlled with the modulation of topical or systemic drug administration.

Increase in IOP is a frequently noted phenomenon after vitreoretinal surgery [15]. Various mechanisms, including gas expansion, anterior chamber inflammation, and iris-lens diaphragm shifting, are suspected causes of acute increase in IOP [15]. In the present study, increase in IOP was the primary reason of treatment plan changes. This was most frequently observed after vitrectomy with tamponade. Similar to previous studies, [8, 16] most increases in IOP were noted within one week after the surgery. Although most cases were well-controlled after administration of IOP-lowering agents, an increase in IOP was the most prevalent reason for prolonged hospitalization. Patients in whom tamponade is planned should be informed that the treatment plan can be changed because of a postoperative increase in IOP.

Sutureless vitrectomy has revolutionized vitreoretinal surgery. Compared to previous technique, it provided more convenient surgery with shortening of operating time and minimizing surgically induced trauma [17]. However, one limitation of this technique is that tight wound apposition is sometimes not made [18]. Therefore, leakage of vitreous fluid from unclosed wound lead postoperative IOP decrease [18]. In some instances, additional procedures, such as sclerotomy sutures, were necessary to control the leakage [12]. In the present study, hypotony was noted in 3 of the patients who underwent vitrectomy and leakage from sclerotomy wound was noted in one of them. Although the incidence was low, discharge was postponed in one-third of patients with hypotony.

Intraocular inflammation is a rare but important complication of vitreoretinal surgeries. In particular, postoperative endophthalmitis is a grave condition that may lead severe vision loss [19]. In the present study, treatment plan was changed in four patients because of intraocular inflammation, and the date of discharge was postponed in three (75.0%) of them for close follow-up.

An interesting finding of the present study is that the surgeon’s experience can influence treatment plan changes. It is well-known that there is a learning curve in vitreoretinal surgery. [20] In the study by Mazinani et al., a learning effect was noted in most surgeons. The learning effect was correlated with the total number of procedures, suggesting the importance of the surgeon’s experience in successful surgery [21]. In a more recent study investigating the redetachment rates after retinal detachment surgery, the rate was approximately 20% in beginners. The redetachment rates steadily decreased and stabilized under 10% after approximately 200 surgeries [20]. In the study of Yamakiri et al., surgeons with a higher average annual number of surgeries had significantly better surgical outcomes in scleral buckling procedure [22]. In the present study, we first evaluated the influence of the surgeon’s experience on the incidence of treatment plan changes. We observed that the incidence was higher in less experienced surgeons than in experienced surgeons, suggesting that inexperienced surgeons should anticipate treatment plan changes before surgery.

The previously reported types and incidence of postoperative complications in patients who had undergone vitreoretinal surgery are summarized in Table 5. The incidence of postoperative complications reported in our study were generally lower than those reportedly [5–25]. However, this study only analyzed cases where treatment plan changes were deemed necessary. Therefore, the incidence of actual complications might have been higher.

**Table 5:** Summary of previously reported types and incidence of postoperative complications following vitreoretinal surgery

Author	Published year	Diagnosis	No. of eyes	Increase in IOP	Intraocular inflammation	Hypotony	Corneal edema
Jabbour et al. [8]	2018	RRD, PDR with tRD, MH	254	48 ~ 61.5% within POD 7	N/A	N/A	N/A
Bamonte et al. [6]	2011	Floater, macular pucker, MH, RD, etc. (VH, retained lens fragments, lens subluxation)	122	N/A	N/A	13.1% on POD 1	N/A
Arikan Yorgun et al. [5]	2016	ERM, VH, MH, RD, VMT	306	15%~25% on POD 1	5 ~ 26% exhibited more than + 2 AC cells on POD 1	1.8 ~ 2.7% on POD 1	N/A
Patel D. et al. [10]	2021	RRD or tRD	418	IOP > 21 mm Hg, 69 eyes (16.5%); IOP > 30 mm Hg, 11 eyes (2.6%)	N/A	N/A	N/A
Moussa et al. [9]	2023	Pseudophakic RRD	456	IOP ≥ 22: 28.0 ~ 49.6%, IOP ≥ 25: 13.6 ~ 30.3% on POD 1	N/A	0 ~ 0.8% on POD 1	N/A
Desai A. et al. [25]	2008	RD, VH, MH, ERM, retained lens fragments, endophthalmitis, floaters	100	2.5 ~ 12% required IOP control on POD 1	N/A	N/A	N/A
CHEN et al. [7]	2016	PDR, MH, ERM, RRD, VH, IOL or lens dislocation, aphakia, SO removal	460	11.5%	N/A	N/A	≥ 1.1%
Ben Ghezala et al. [24]	2020	ERM, MH	152,034	N/A	0.25% for suspected endophthalmitis	N/A	N/A
AL-Hinai et al. [23]	2017	N/A	121	N/A	N/A	N/A	0.80%

Abbreviations: *IOP *Intraocular pressure, *RRD *Rhegmatogenous retinal detachment, *PDR *Proliferative diabetic retinopathy, *tRD *tractional retinal detachment, *MH *Macular hole, *POD 7 *Postoperative day 7,  *N/A *Not available, *RD *Retinal detachment, *VH *Vitreous hemorrhage, *POD 1 *Postoperative day 1, *ERM *Epiretinal membrane, *VMT *Vitreomacular traction, *AC *Anterior chamber, *IOL *Intraocular lens, *SO *Silicon oil

The strengths of the present study are as follows. Firstly, we first evaluated the incidence of changes in the duration of hospitalization after vitreoretinal surgery owing to treatment plan changes. Although hospitalization was prolonged in only a limited number of patients, the postoperative care plan and subsequent cost of treatment could be markedly changed in these patients. Recently, the number of centers accredited by the Joint Commission International has increased worldwide, [26] suggesting an increasing need for standardization of patient care. In the treatment of a certain disease, predicting the hospitalization period is an important part of establishing a standardized care plan for a patient. Second, we identified incidence, timing, and factors related to changes in treatment plans, which can provide valuable information for establishing treatment plans and discussing them with patients in advance.

The present study had some limitations. Firstly, this retrospective study was conducted at a single institution. Secondly, since patients with certain diagnoses were included, the results of the present study may not be valid for patients who underwent vitreoretinal surgery for other reasons. Thirdly, analysis was performed based on the data obtained within 2 weeks after surgery. Thus, complications that may occur after a longer period, such as open-angle glaucoma, [27] could not be evaluated. Fourth, no strict guidelines were available for surgery, and our results included surgeries performed by multiple surgeons. Therefore, the results of this study require confirmation via further studies with a more controlled design. Finally, detailed postoperative anatomical or functional changes [28] were not evaluated. Subsequent evaluations may be necessary to assess the impact of such changes on the risk of treatment plan changes.

In summary, treatment plan were changed due to postoperative complications in 16.3% of patients within 14 days following vitreoretinal surgery. The most prevalent complication was an increase in IOP. The incidence was higher in patients who underwent gas or oil tamponade and those who underwent surgery performed by less experienced surgeons. In some patients, the discharge date was delayed because of changes in the treatment plan. When establishing a standardized care plan for patients requiring vitreoretinal surgery, the possibility of treatment plan changes and subsequent prolongation of hospitalization should be considered. Further studies are needed to elucidate the specific burdens that treatment plan changes may impose on individual patients.

## Data Availability

The datasets generated during and/or analyzed during the current study are available from the corresponding author upon reasonable request.
